# 470-Million-year-old black corals from China

**DOI:** 10.1007/s00114-012-0947-8

**Published:** 2012-07-12

**Authors:** Andrzej Baliński, Yuanlin Sun, Jerzy Dzik

**Affiliations:** 1 Instytut Paleobiologii PAN, Twarda 51/55, 00-818 Warszawa, Poland; 2 Key Laboratory of Orogenic Belts and Crustal Evolution, School of Earth and Space Sciences, Peking University, Beijing, 100871 China; 3 Instytut Zoologii Uniwersytetu Warszawskiego, Banacha 2, 02-097 Warszawa, Poland

**Keywords:** Anthozoa, Phylogeny, Fossil record, Molecular clock, China

## Abstract

Phosphatic (possibly secondarily phosphatised) remains of antipatharian coralla, previously unknown in the fossil record, occur abundantly in the early Ordovician Fenxiang Formation in the Hubei Province, southern China. Probably two species (and genera) are represented, which differ in spinosity of branches. The more spinose one, *Sinopathes reptans*, has its lateral spines bearing regular, longitudinally arranged costellae. The early Floian geological age of this finding, about 470 Ma, supports predictions on the timing of anthozoan phylogeny derived from the molecular phylogenetic evidence. Black corals (Antipatharia) are basal to the scleractinians in the Hexacorallia clade, being more derived than sea anemones and the Zoantharia. Based on calibration of the molecular clock with Mesozoic data, the first split of lineages within the scleractinian hexacorals was proposed to take place approximately 425 million years ago. This implies that the origin of Antipatharia should precede this date. They have not been known in the fossil record because of unmineralised skeleton composed primarily of laminar chitin complexed with a protein. Unlike all recent species, the encrusting basal part of the colony dominated in the Ordovician ones and only occasionally erect branches developed, rather chaotically ramified. This presumably plesiomorphic trait seems consistent with ancient geological age and suggests that some problematic fossils from the Late Cambrian may be their, even less-derived, relatives.

## Introduction

**Fig. 1:**
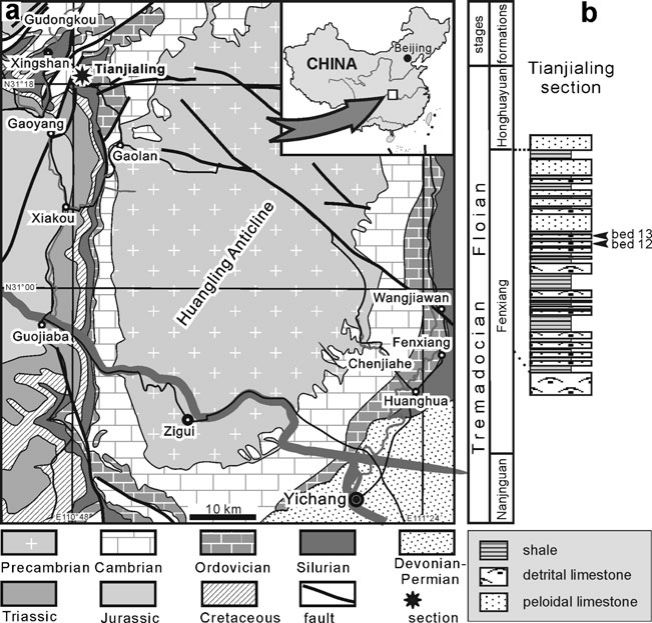
Map and geological section showing the geographic location and stratigraphic position of the fossil site (Fenxiang Formation). **a** Geological sketch map of Yichang area, Hubei Province, China and **b** position of sampled beds in the rock column at the Tianjialing section

Black corals (Antipatharia), highly prized in jewellery and on the list of endangered species, occupy a crucial location on the anthozoan phylogenetic tree. In the Hexacorallia clade, they are basal to the scleractinians, being more derived than sea anemones and the Zoantharia (Brugler and France 2007; Sinniger and Pawlowski 2009). Recent estimates suggest that the first split of lineages within the scleractinian hexacorals took place approximately 425 million years ago (Stolarski et al. 2011). This means that the antipatharian lineage emerged much earlier. However, until recently, they have not been known in the fossil record due to an unmineralised skeleton composed primarily of laminar chitin in a complex with the protein antipatharin (Goldberg 1991; Williams et al. 2006) to form a kind of fibrillar plywood structure (Kim et al. 1992). Structural chitin does not preserve in older rocks unless transformed in aliphatic compounds (Gupta and Summons 2007), but it commonly phosphatises (e.g. Doguzhaeva and Mutvei 2006). This could have been the case with the fossil antipatharians extracted from limy intercalations within the middle part of the Fenxiang Formation exposed near the village of Tianjialing (Fig. 1) in the Yichang area (also known as Three Gorges area) of Hubei Province, located at the centre of the Yangtse Platform (Zhan and Jin 2007). The external morphology of spinose branches and the lamellar structure of the skeleton of the Fenxiang fossils are closely similar to the present-day antipatharians. These early antipatharians encrusted various substrates, e.g. bryozoan colonies and monaxonian sponges. Early diagenetic mineralisation of the Chinese fossils may have been promoted by incipient phosphatisation that is known to occur also in skeletons of extant gorgonarians. It was suggested that this “may indicate a previous history of phosphatic skeletal mineralisation within this phylum” (Macintyre et al. 2000).

The Ordovician Fenxiang (transcribed also Fenhsiang) Formation consists mainly of dark grey to grey skeletal and peloidal limestone and greenish grey shale, about 40 to 50 m thick in the east and about 20 m thick in the west of the anticline. All the studied material has been collected from the middle part of the Fenxiang Formation near the village of Tianjialing. The section at Tianjialing is located on the northwest edge of the Huangling Anticline and about 8 km southeast of the Xingshan County (Fig. 1).

The antipatharians are not the only phosphatic fossils in the Fenxiang Formation (Long and Burrett 1989). The formation is renowned for its invertebrate fossils, including the oldest bryozoan–lithistid sponge framework (Zhu et al. 1995; Xia et al. 2007; Adachi et al. 2011), as well as pyritised originally organic hydrozoan colonies and linguloid brachiopods preserving soft parts with remarkable fidelity. In the Tianjialing section, trepostome and rhabdomesonate bryozoans, trilobites and benthic graptolites (*Acanthograptus* and *Koremograptus*, A. Kozłowska personal communication 2012) are the most common fossils. In acid-resistant residues, linguloid brachiopods, conodonts and phosphatised remnants of phyllocarid crustaceans (e.g. setae) co-occur with the antipatharians. Conodont specimens (77) extracted from sample 13 represent a low-diversity assemblage dominated by an unidentified generalised species of *Drepanodus*, a species of *Drepanoistodus* probably conspecific with that occurring in the Emanuel Formation of Australia (Zhen and Nicoll 2009), and *Acodus triangularis* (Ding in Wang, 1993). The latter has finely denticulated processes (Zhen et al. 2005), which means that the age of the source bed is early Floian (Arenig in British terms). It is younger than the base of the *Tetragraptus approximatus* Zone at Hunneberg in Sweden, the stratotype for the boundary between the Tremadocian and Floian (Löfgren and Bergström 2009) where undenticulated *Acodus deltatus* Lindström, 1955 (a chronospecies representing the same lineage) occurs.

## Materials and methods

The studied specimens were extracted from two samples (taken from beds 12 and 13) of limy shale, weighting approximately 7 and 10 kg, respectively, which were dissolved in diluted acetic acid. More than 700 specimens were picked out from the residua, and about 160 of them have been mounted on the SEM stubs, sputter coated with carbon and platinum, and studied under the Phillips XL-20 scanning electron microscope equipped with EDS detector ECON 6, at the Institute of Paleobiology, Warsaw, Poland.

Three species of the recent antipatharian corals have been examined. To expose internal structure of the skeleton, pieces of the stem were treated with concentrated formic acid for several hours at room temperature. Fragments of branches of *Parantipathes larix* with polyps were dehydrated in a graded ethanol series samples, dried in the critical point dryer, and sputter coated with carbon and platinum for SEM.

## Systematic palaeontology

Cnidaria Hatschek, 1888

Anthozoa Ehrenberg, 1834

Hexacorallia Haeckel, 1966

Antipatharia Milne Edwards, 1850

*Sinopathes reptans* gen. et sp. nov.

### Holotype

**Fig. 2:**
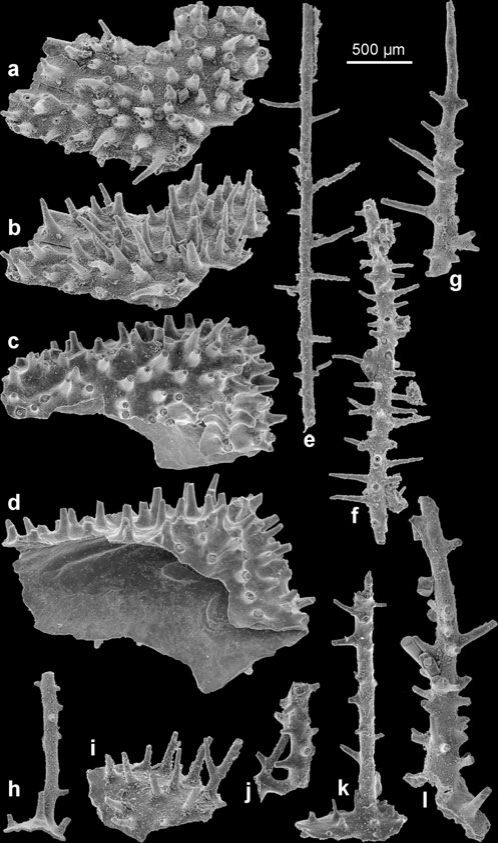
The Early Ordovician phosphatic skeleton of *S. reptans* gen. et sp. nov., showing variable disposition of spines on encrusting and erect parts of the corallum. **a**, **b** Flat basal part of a colony showing crowded spines, viewed vertically from the exterior and oblique laterally (holotype ZPAL H. 27/6-7); **c**, **d** fragment of spinose encrusting part of colony in external and oblique-lateral views (ZPAL H. 27/8-13); **e**, **g** erect branches showing disposition of spines (ZPAL H. 27/2-16, 6-6, 5-17); **h**, **k**, **l** three specimens showing partly preserved spinose basal part of colony and erect spinose branches (ZPAL H. 27/18-25, 8-1, 8-9); **i** flat, basal part of colony with numerous spines, note one bifurcating spine (ZPAL H. 27/10-3); and **j** piece of colony branches that cross and merge (ZPAL H. 27/8-7)

**Fig. 3:**
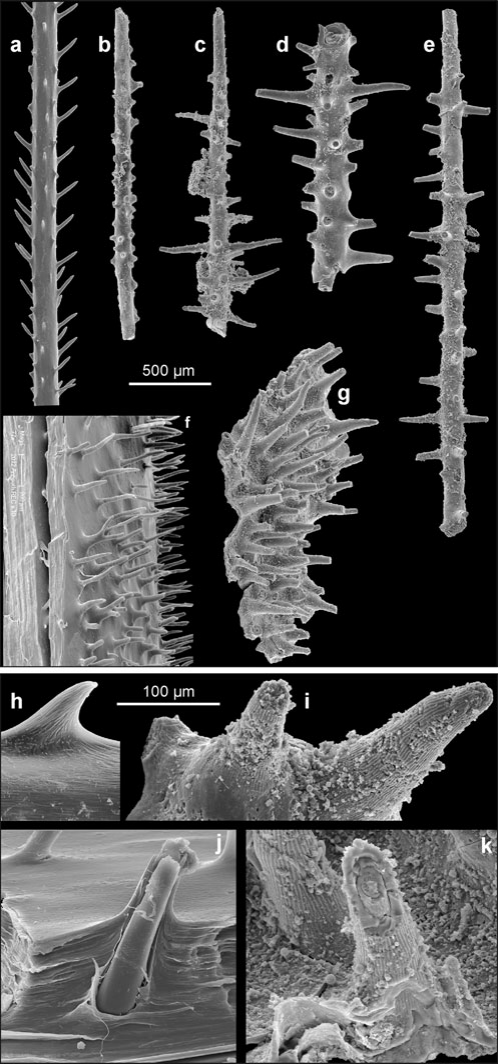
The Early Ordovician phosphatic skeleton of *S. reptans* gen. et sp. nov., compared with coralla of extant antipatharians. **a** Lateral branch of the extant *A. subpinnata* (Ellis and Solander, 1786) from the Strait of Messina (Tyrrhenian Sea) (ZPAL H. 27/16-1); **b**–**e** erect branches of *S. reptans* (ZPAL H. 27/8-3, 5-18, 6-1, 18-27); **f** spinosity on the main branch of *A. subpinnata* (ZPAL H. 27/17-2); **g** spinosity on the encrusting part of the colony in *S. reptans* (holotype ZPAL H. 27/6-7); **h** surface ornamentation on pinnular spine in the extant *P. larix* (Esper, 1790) of unknown origin (specimen from the old museum collection of the University of Warsaw) (ZPAL H. 27/13-2); **i** ornamentation of spines in *S. reptans* (ZPAL H. 27/5-20); **j** transverse section of the main branch of *A. subpinnata* showing its lamellar structure (ZPAL H. 27/17-2); and **k** lamellar structure of the corallum of *S. reptans* (ZPAL H. 27/7-21)

ZPAL H. 27/6-7 (Figs. 2a, b and 3g), partial corallum associated with several hundreds of more or less fragmentarily preserved branches and basal parts of coralla in sample XS 13U.

### Locality

Exposure near the village of Tianjialing in the Yichang area (also known as Three Gorges area) of Hubei Province.

### Horizon

Upper part of the Ordovician Fenxiang Formation (early Arenigian).

### Diagnosis

Antipatharian with extensive encrusting basal part of phosphatic (perhaps early diagenetically phosphatised) corallum, and thin erect branches that are covered with slender spines, rather disorderly distributed. Spines are ornamented with sharp parallel longitudinal delicate costellae, approximately 1 μm wide and 3–4.5 μm apart. Axes of erect branches are straight and cylindrical.

### Description

**Fig. 4:**
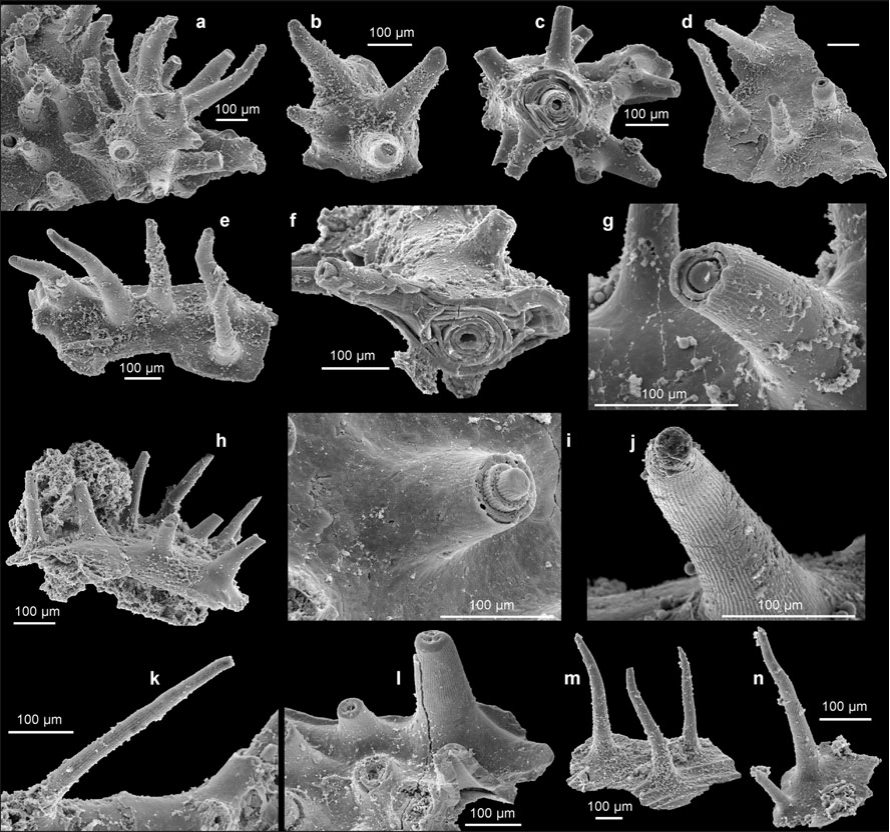
Morphology and structure of spines in phosphatic coralla of *S. reptans* gen. et sp. nov. from the Early Ordovician Fenxiang Formation exposed near the village of Tianjialing in the Hubei Province, China. **a**, **b**, **d**, **e**, **h**, **m**, **n** Fragments of the basal (encrusting) part of coralla showing variability in spinosity (ZPAL H. 27/7-21, 5-20, 18-29, 2-1, 1-24, 2-4, 2-12, ); **c**, **f** pieces of major branches with ramifications showing surface spinosity and lamellar structure at the transverse fracture (ZPAL H. 27/8-7, 8-5); **g**, **i** enlargements of spines showing longitudinal costellation and lamellar structure at their broken tips (ZPAL H. 27/6-6, 8-13); **j** spine with well-preserved longitudinal costellation (ZPAL H. 27/6-1); **k** long and slender spine with longitudinal costellation (ZPAL H. 27/1-5); and **l** fragments of the basal (encrusting) part of corallum showing costellate spines, note a distally narrowing central lumen in the broken spine (ZPAL H. 27/3-19)

The colonial skeleton of *S. reptans* and associated unnamed antipatharian species consists of two kinds of morphologically different elements: the encrusting basal unit and ascending branches. The larvae apparently settled on hard objects resting on the muddy sea bottom. The basal unit grew over the substrate, and it is usually irregular, from almost flat to tube-like, occasionally wrinkled. Its lower surface precisely replicates the substrate morphology (Figs. 2d and 4a, k).

**Fig. 5:**
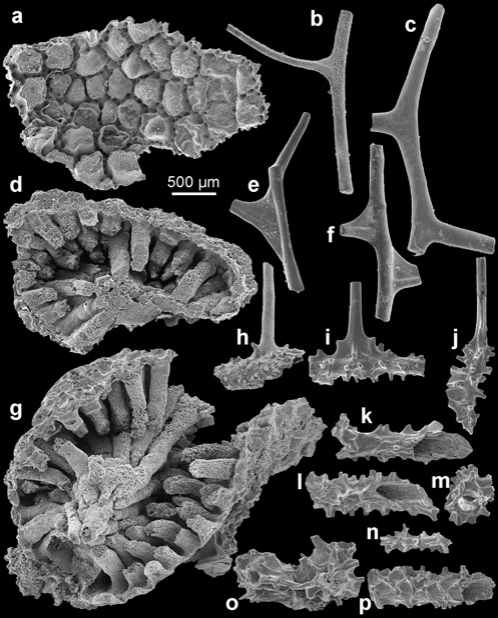
Unnamed antipatharian from the Fenxiang Formation showing variable disposition of spines on basal and erect parts of the corallum. **a** Phosphatic coating of a bryozoan zoarium replicating its external surface in detail, the coating is encrusted on its external surface by an antipatharian colony (ZPAL H. 27/10-12); **b**, **c**, **e**, **f** pieces of colony branches (ZPAL H. 27/6-14, 5-15, 7-10, 6-19); **d**, **g** two specimens of bryozoan zoaria encrusted from the exterior by antipatharians, note the siliceous infillings of bryozoan zooecia (ZPAL H. 27/10-10, 10-11); **h**–**j** fragments of tubular spinose encrusting colony of antipatharians with smooth lateral branches (ZPAL H. 27/18-7, 7-8, 18-5); and **k**–**p** fragments of tubular spinose encrusting colonies of antipatharians (ZPAL H. 27/7-7, 8-17a, 8-17b, 5-19, 8-12, 5-12)

In most cases, the lower surface of the basal unit is smooth or too indifferent to enable identification of the substrate. However, in several specimens of both Fenxiang species, it reproduces, in negative, bryozoan zoaria (Fig. 5a), sponge cortical spicules (Fig. 5k), or trilobite skeletal parts. The most common are specimens attached to bryozoan colonies. In some cases, the only material remaining after the zoarium is a replica of its surface morphology in the corallum (Fig. 5a). Frequently, the zooecia are partially filled with sediment cemented by phosphate or silica, and the whole zoarium is covered with a diagenetic phosphatic lining (Fig. 5d, g). There is a great variety of bryozoans in the sample, but their fragmentary preservation precludes identification at species or even a generic level. Judging from the zoarial shapes and spinose zooecial apertures, both trepostomes and rhabdomesonates are present there. The bryozoans are frequent fossils in the Fenxiang Formation, where they occasionally formed reefs (Adachi et al. 2011, 2012). Phosphatic linings of bryozoans from this locality have been already described as possible ascidians under the name *Fenhsiangia zhangwentangi* (Long and Burrett 1989). Bryozoans preserved in this way are not uncommon in the Ordovician (e.g. Dzik 1994).

In a few antipatharian specimens from the Fenxiang Formation, the basal surface replicates the morphology of monaxonic sponge spicules (oxeas) in parallel arrangement (Fig. 5k). Apparently, the antipatharian colonies were growing on thin tubular projections of sponges with parallel arrangement of spicules. Such spicules easily disintegrate, and no articulated sponge of comparable anatomy is known from the early Palaeozoic (Reitner and Wörheide 2002). Some Silurian species showing parallel arrangement of monaxonic spicules may be related to this one (Muir and Botting 2007).

The upper surface of the basal unit of *S. reptans* is densely covered with erect and rather slender spines, relatively long as compared with extant antipatharians. Density of spines varies from 25 to 53 per 1 mm^2^ with an average of about 40 spines per 1 mm^2^. The spines have a wider, conical and smooth proximal part and long, slimmer distal part with narrowing, but bluntly rounded apex (Figs. 3i and 4j). At their bases, the spines attain 40–140 μm in diameter, but they narrow to 13–30 μm near their tips. Length of the spines is 330–600 μm. They are more or less straight or slightly inclined to bend. Rarely, they bifurcate (Fig. 2i).

Apart from their basal part and apex, all surfaces of spines in *S. reptans* are sculptured with delicate but sharp, longitudinal, 1-μm-wide ridges or costellae. The costellae are spaced every 3.0–4.5 μm and usually run more or less parallel to each other, but occasionally, they may bend slightly, intercalate or bifurcate (Figs. 3i *,* k and 4g, j). The spines are narrowly conical in shape, with particular laminae stacked one above the other (Fig. 3k) in result of their secretion from outside. The characteristic surface ornamentation is well visible also on the internal (adaxial) layers in broken spines (Figs. 3k and 4i).

Some of the spines on the encrusting part of the colonial skeleton may be more robust than others, and they branch or develop lateral spines. These are probably incipient erect parts of the colony. It is not clear whether more than one erect branch developed from single base, but this seems likely because the branching spines do not show any preferred location.

Erect branches are straight to slightly curved (Figs. 2e–h, k, l and 3b, c, e, f). The diameter of branches ranges from 40 to 360 μm. Their wide central lumen may be markedly reduced in massive, thickened specimens with multilayered structure (Fig. 4c, f). Branches are covered with spines of the same type as those covering the basal unit. The spines are distributed rather irregularly and with variable density, significantly lower on thinner branches. The spines are slightly inclined adapically on some branches. Bifurcation of spines, similar to that on the basal surface, also occurs. Branches of diameter less than 70 μm are usually devoid of spines (Fig. 2g). Second order lateral branches arise occasionally at about a right angle or obliquely relative to the lower order branch. There may have been branching of a few orders. Occasionally, the lateral branches may touch the other ones and merge, forming a complex rigid network (Fig. 2j).

**Fig. 6:**
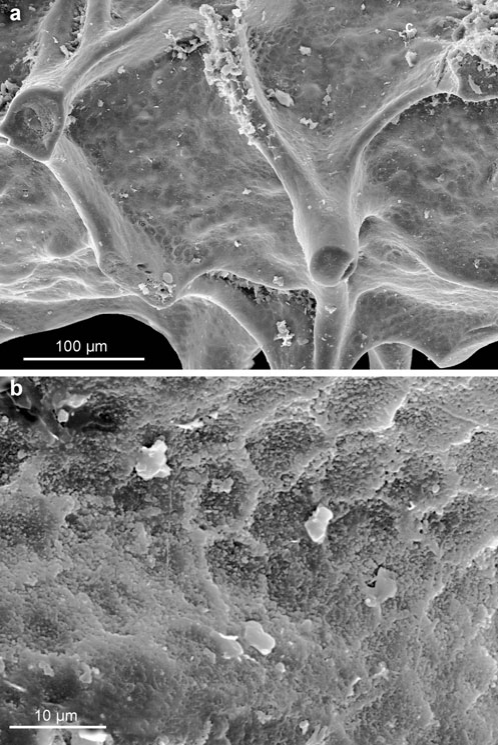
Unnamed antipatharian from the Fenxiang Formation showing imprints of secretory epithelial cells: **a**, **b** specimen ZPAL H. 27/15-2

The unnamed antipatharian associated with *S. reptans* frequently shows imprints of secretory epithelial cells (Fig. 6). They are especially well developed in depressions between basal ribs of the spines. Their size is generally uniform ranging from 6 to 9 μm. They resemble imprints left on the phosphatic skeleton by secretory epithelial tissue in brachiopods (Williams and Cusack 2007) or conodonts (Conway Morris and Harper 1988; von Bitter and Norby 1994; Dzik 2000). Unlike similar size epithelial cells contributing to the skeleton in arthropods (Olempska 2011) or the Cambrian lapworthellid problematica (Balthasar et al. 2009), the cells were not incorporated into the skeleton, imprints being concave, not convex. The concavities apparently reflect only shapes of their basal secretory surfaces.

## Results and discussion

*S. reptans* is closely similar to present-day antipatharians. No fossil or recent organisms of other taxa match its morphology. The extant antipatharians are a well-defined group of about 230 known species of clonal colonial hexacorals classified in 42 genera and seven families (Opresko in Daly et al. 2007; Lapian et al. 2007). The ramose colonies are attached to the substrate with a strong stem, but the elevated part of the colony varies within the group. Their organic skeleton of black or brownish colour gave them their common name of black corals. The antipatharian skeleton is completely covered with soft tissue that secretes it basally and shows little correspondence to the anatomy of the polyps. Its unique, as for the corals, aspect is the presence of numerous spines (thorns) arising from the skeleton surface.

The spine density in *S. reptans* measured on the encrusting part of the colonial skeleton ranges from 25 to 52 per mm^2^ and falls well in the range of measurements for the extant antipatharians (Fig. 3f). Spines covering their main axes as well as branches and pinnules are very variable between species in their distribution density, growth pattern, size, shape and surface ornamentation. For instance, in the schizopathid *P. larix*, the spine density measured on the stem surface and largest branches is 8–9 per mm^2^. In the myriopathids *Antipathella subpinnata* and *Myriopathes panamensis*, the spines are more densely crowded being 32–40 and 48–55 per mm^2^, respectively. Also, the length of spines is variable. In some schisopathids, spines on the pinnules are 20–60 μm tall (e.g. Opresko 2002); in *P. larix*, they are 50–70 μm tall. In the myriopathids they may reach 700 μm in length. This is comparable to the spines in *S. reptans* that are 330–600 μm long. Among recent antipatharians, those with the most spinose appearance seem to occupy the basal position in their phylogenetic tree (Lapian 2009).

In extant antipatharians, spines are often arranged in longitudinal rows and tend to be inclined distally (Fig. 3a). This does not refer to *S. reptans*, in which spines are rather chaotically distributed and vary in size and shape. This condition seems to be plesiomorphic. However, there is a tendency to adapical inclination of spines on branches. In some extant black corals, the tips of the pinnules lack spines. Opresko (2002) interpreted this as a result of the sclerenchyme growing in spurts. Also, in *S. reptans*, the thinnest distal parts of the branches are barren of spines (Fig. 2g).

In some recent antipatharians where there are spines of distinctly different sizes, the longest spines are often confined to the polyp side of the axis or the longest spines occur on the periphery of the polyps (which are confined to one side of the axis). Such correlation between the size of the spines and the position of the polyps (Opresko 2001, 2002, 2006) does not appear to be the case for the described fossil, which would argue for a rather random placement of the polyps.

*S. reptans* differs from all extant antipatharians and from associated unnamed species from the Fenxiang Formation in the surface ornamentation of spines. The spines are invariably sculptured with delicate costellae, irrespective of the spine size or shape, both in the encrusting and erect parts of the colony. The pattern of spinosity varies greatly, and elongation of spines (Figs. 3i, k and 4g, j) is also very variable. None of the recent antipatharians possess spines with so regular ornamentation although several species do have them longitudinally ribbed (Fig. 3h). Similar in general appearance, albeit slightly larger, longitudinal costellae occur on anterior pinnules in the cladopathid *Heliopathes americana* (Opresko 2003, Fig. 19a, b). Some of the costellae extend from the surface of pinnules onto their spines. Thus, although the costellation on spines in the Ordovician form seems to be very specific, a similar ornament can be found in extant antipatharians, as well.

The corallum of extant antipatharians has a laminar structure and reveals tightly apposed concentric laminae interspersed by radially oriented spines (Goldberg 1991; Kim et al 1992). This skeletal framework is well revealed in the delaminated specimen of *A. subpinnata* (Fig. 3f, j, see also Goldberg 1991), in which consecutive growth layers cover all surface of the skeleton including spines. The mode of growth of the skeleton and the structural framework of *S. reptans* are virtually identical (Figs. 3k and 4c, f). Transverse sections of the basal plate of *S. reptans* reveal different thickness of laminae. Thin laminae are 1–3 μm thick and have a compact structure. They interlayer with laminae 5–40 μm thick, which appear to be less compact and rather “baculate”. Such structure can be found, e.g. in the recent *Antipathes fiordensis* (Goldberg 1991, Fig. 1) and may be a seasonal growth pattern.

Unlike extant antipatharians, in *S. reptans*, the basal encrusting unit of the corallum dominates over the erect part. This may also be a plesiomorphic condition. Although some recent black corals fix to the substrate with hooked basal portion of the stem inserted in the soft sediments, most of them cement themselves by dilated basal disc encrusting the hard substrate (Pasternak 1977). An example is the corallum of *Antipathes aperta*, up to 4.6 m tall, that arises from a large, encrusting rocky substrate holdfast that in old specimens spreads for up to 40–60 cm^2^ and is covered with spines (Grange 1988).

Phosphatic (probably phosphatised) coralla are known in fossil Early Palaeozoic octocorals. Lindström (1978) reported such composition of a ramose corallum of the probable gorgonian *Nonnegorgonides ziegleri* from the Ordovician (Floian, about 470 Ma) limestone of Sweden. *Nonnegorgonides* specimen is much larger and a little geologically younger than *Sinopathes* and may be more advanced either in terms of evolution or ontogeny. Ramose skeletons with diagenetically altered composition resembling the present-day gorgonians occur also in the Arenig strata of Wales (Cope 2005). *Palaeobotryllus taylori* from older, Late Cambrian, strata of Nevada is another fossil with laminated phosphatic structure and covered with spines (Müller 1977). Its skeleton forms chambers with completely smooth upper surface and growth increments visible on flanks. This shows that it was secreted basally by a continuous soft tissue cover, with spines being also covered with epithelium. Chambers give *Palaeobotryllus* a little more corallum-like appearance than *Sinopathes*, and they may represent rudimentary corallites.

**Fig. 7:**
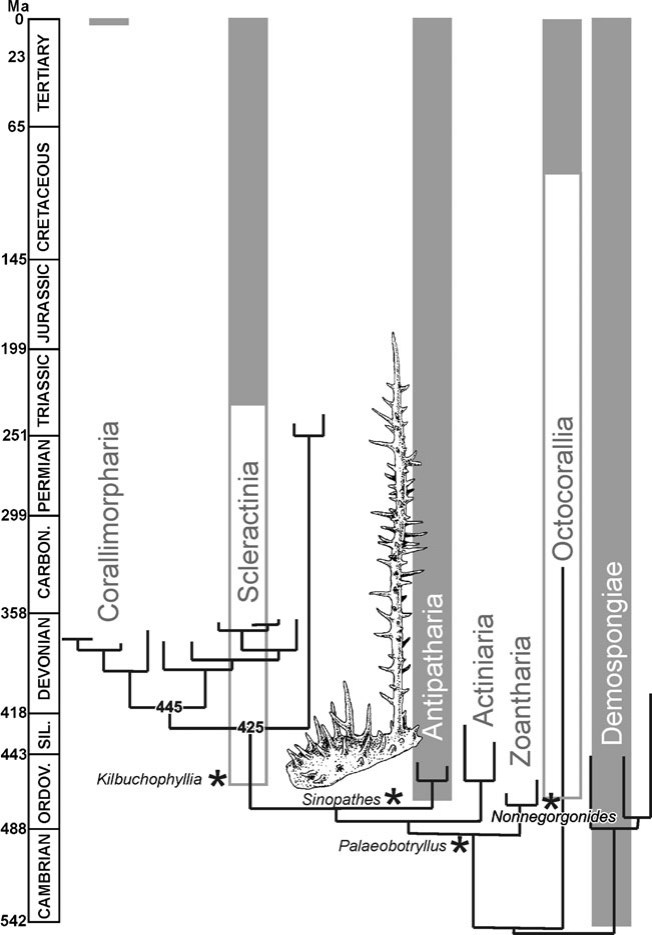
Anthozoan phylogenetic tree based on mitochondrial protein coding genes (Sinniger and Pawlowski 2009) calibrated with Fenxiang antipatharian and with molecular clock extrapolated from the Mesozoic scleractinian fossil record (Stolarski et al. 2011; note that Corallimorpharia were there used as outgroup and this resulted in their basal position in respect to the Scleractinia). Unquestionable fossil evidence indicated in *grey*, disputable in *white*

Although it remains undecided whether *Sinopathes* is originally or secondarily phosphatic, its morphology strongly suggests affinity to the extant black corals. Despite their basal location on the molecular phylogenetic tree, the antipatharian skeleton is highly derived. This refers also to *Sinopathes*, even if the basal encrusting part of the colony seems to dominate over the erect branches. Derived condition of morphology in an Ordovician member of the group indicates even more ancient origin of the lineage. This well agrees with estimates inferred from the molecular clock (Fig. 7) calibrated with the scleractionian fossil record (Stolarski et al. 2011). The discovery of antipatharians in the Early Ordovician calls for evaluation of earlier findings of phosphatic fossils with problematic affinities. This may allow the gap between the earliest cnidarians without skeleton and the skeletonised corals to be filled.
